# External fixation versus elastic stable intramedullary nailing in the treatment of open tibial shaft fractures in children

**DOI:** 10.1186/s13018-021-02679-w

**Published:** 2021-08-25

**Authors:** Pan Hong, Saroj Rai, Xin Tang, Ruikang Liu, Jin Li

**Affiliations:** 1 Department of Orthopaedic Surgery, Union Hospital, Tongji Medical College, Huazhong University of Science and Technology, Wuhan, China; 2 Department of Orthopaedics and Trauma Surgery, Blue Cross Hospital, Tripureswor, Kathmandu, 44600 Nepal; 3 First Clinical School, Tongji Medical College, Huazhong University of Science and Technology, Wuhan, China

**Keywords:** External fixator, Open tibial fracture, Elastic stable intramedullary nail

## Abstract

**Introduction:**

External fixator (EF) is a popular choice for open tibial fractures, but pin tract infection (PTI) and refracture are common complications. Elastic stable intramedullary nail (ESIN) has been reported in the treatment for open tibial fractures. This study aims to compare the clinical outcomes of EF vs. ESIN in the treatment for open tibial shaft fracture in children retrospectively.

**Methods:**

Patients aged 5–11 years with Gustilo-Anderson II and IIIA tibial shaft fracture treated at our institute from January 2008 to January 2018 were reviewed retrospectively and categorized into EF and ESIN groups. Patients with pathological fracture, neuromuscular disorder, metabolic disease, previous tibial fracture or instrumentation, and polytrauma were excluded. Patients with follow-up < 24 months or incomplete medical records were also excluded.

**Results:**

In all, 55 patients (33 males, 22 females) were included in the EF group, whereas 37 patients (21 males, 16 females) were included in the ESIN group. There was no statistically significant difference between the two groups concerning sex, age, body weight, duration from injury to surgery, Gustilo-Anderson (GA) classification, and concomitant injuries. There was no case of nonunion and malunion in either group. The angulation at the latest follow-up was higher in the EF group than the ESIN group (*P* < 0.01). The radiological union was faster in the ESIN group (7.0 ± 0.9 weeks) than those in the EF group (9.0 ± 2.2 weeks) (*P* < 0.01). Limb length discrepancy (LLD) was more in the EF group (12.1 ± 4.4, mm) than in the ESIN group (7.3 ± 4.3, mm) (*P* < 0.01).

**Conclusion:**

ESIN is a viable option in selected patients of GA grade II and IIIA open tibial fractures with comparable clinical outcomes as external fixator. Pin tract infection is the most troublesome complication in the EF group while implant prominence is a nuisance in the ESIN group.

## Background

Tibial fracture is a common injury in children, and it usually involves the diaphysis and distal metaphyseal region [1]. Closed reduction followed by a well-molded casting remains the primary choice for closed tibial shaft fracture [2, 3]. However, for some fractures such as comminuted or unstable fractures, open fractures, and polytrauma, surgical stabilization is usually warranted [4–6]. Although Charalambous et al. reported debridement followed by casting vs. surgical fixation [7], utilization of external fixator (EF) remains a popular choice for open injuries. However, pin tract infection (PTI) and refracture are common complications during the application of EF [8, 9]. Besides, elastic stable intramedullary nail (ESIN) has also been reported with an acceptable outcome for the treatment of open tibial fractures [10, 11].

This study aims to compare the clinical outcomes of EF vs. ESIN in the treatment for open tibial shaft fracture in children retrospectively.

## Methods

**Fig. 1:**
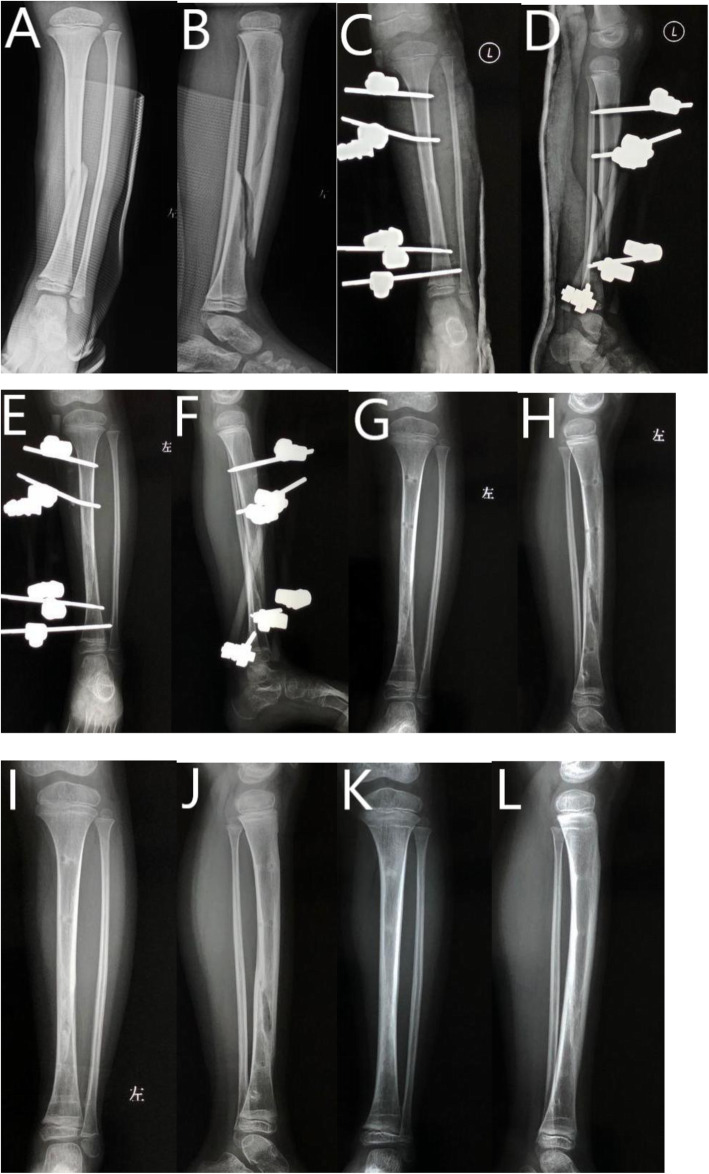
Six-year-old boy with Gustilo-Anderson grade II tibial fracture treated with EF. **A** AP view of tibia before surgery. **B** Lateral view of tibia before surgery. **C** AP view of tibia after surgery. **D** Lateral view of tibia after surgery. **E** AP view of tibia at 8th week follow-up. **F** Lateral view of tibial at 8th week follow-up. **G** AP view of tibia after hardware removal at 11th week follow-up. **H** Lateral view of tibia after hardware removal at 11th week follow-up. **I** AP view of tibia at 5th month follow-up. **J** Lateral view of tibia at 5th month follow-up. **K** AP view of tibia at 12th month follow-up. **L** Lateral view of tibia at 12th month follow-up

**Fig. 2:**
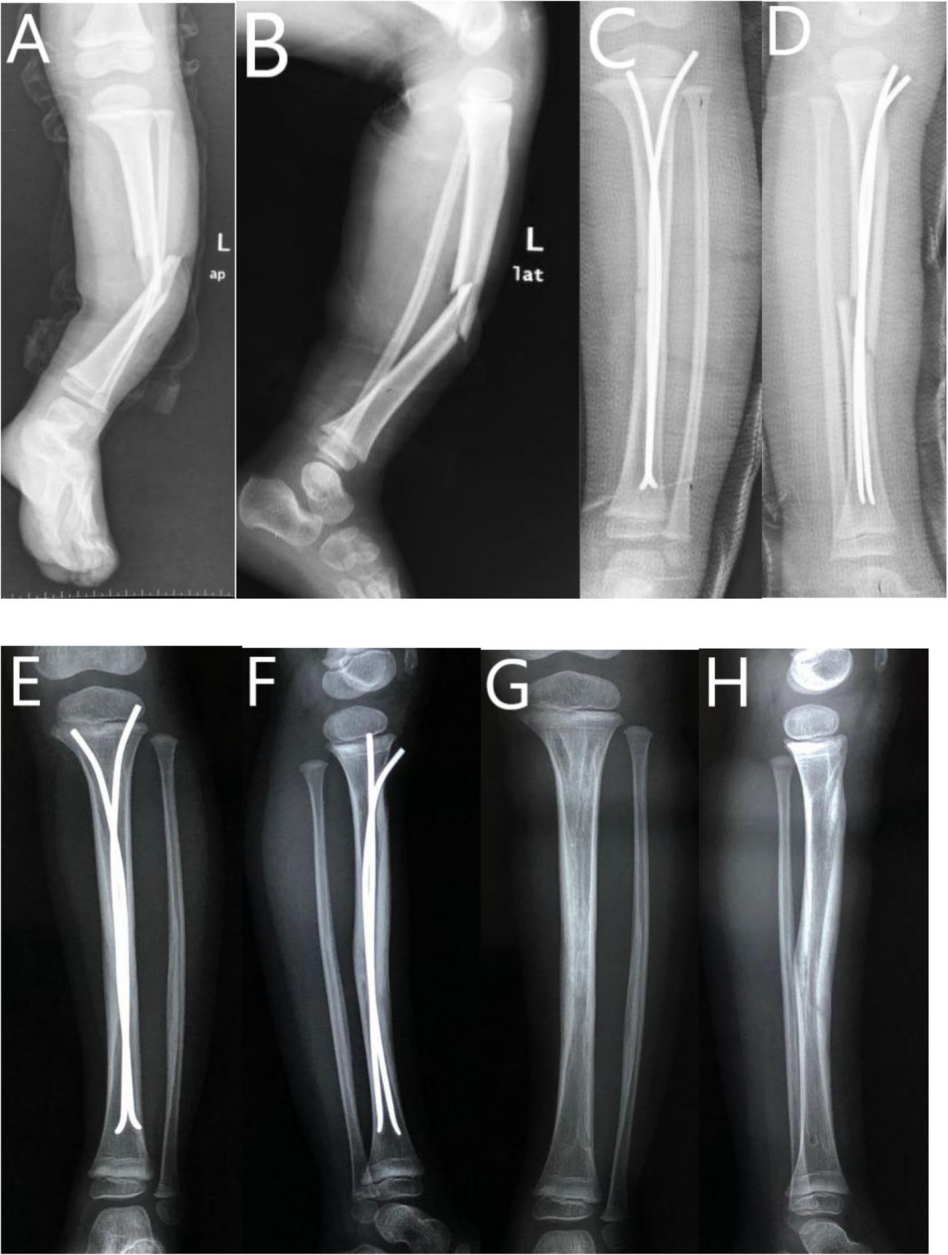
Five-year-old girl with Gustilo-Anderson grade IIIA tibial fracture treated with ESIN. **A** AP view of tibia before surgery. **B** Lateral view of tibia before surgery. **C** AP view of tibia after surgery. **D** Lateral view of tibia after surgery. **E** AP view of tibia at 5th month follow-up. **F** Lateral view of tibia at 5th month follow-up. **G** AP view of tibia after hardware removal. **H** Lateral view of tibia after hardware removal

Patients aged 5–11 years with open tibial shaft fracture treated at our institute from January 2008 to January 2018 were reviewed retrospectively. They are categorized into EF and ESIN groups as per their surgical procedure. Gustilo-Anderson (GA) classification was adopted to stratify the patients with open injuries [12]. Patients with GA grade I injuries were excluded from this study. Patients with comminuted fracture, pathological fracture, neuromuscular disorder, metabolic disease, previous tibial fracture or instrumentation, and polytrauma were excluded. In order to monitor the limb length discrepancy (LLD) after surgery, patients with a follow-up period of < 24 months or incomplete medical records were also excluded. Patients with a bodyweight over 50 kg were excluded because ESIN was not adopted for these patients in our hospital (Figs. 1 and 2).

The surgical choice depended on the preference of the surgeon in charge. Antimicrobial therapy included early administration of the first-generation cephalosporin, with or without linezolid, and was dependent upon the contamination and drug sensitivity test. ESIN is routinely removed at approximately 6–8 months in our center.

A full-length anteroposterior (AP) radiograph was used to determine the total length of the tibia. Significant LLD was defined as a difference of at least 2 cm between limbs on a radiograph at the latest follow-up. Angulation was measured as an angle between the anatomic axes of the proximal and distal fragments, and significant angular deformity was defined as coronal angulation > 5 degrees or sagittal angulation > 10 degrees at the latest follow-up. Radiographic union was defined as the appearance of bridging callus across the fracture site on at least 3 out of 4 cortices on AP and lateral radiograph.

Complications were categorized into major and minor ones. Major complications included deep infection, nonunion, or loss of reduction requiring repeated surgery. Minor complications included minor LLD (less than 2 cm) or angular deformity, implant prominence, and superficial infection.

Short leg slab was used to immobilize the operated leg for 3–4 weeks postoperatively. Follow-up was scheduled at 1, 2, 3, 6, 9, and 12-month postoperatively and annually thereafter. Full-length radiograph of the tibia was routinely performed in our hospital during each out-patient visit.

This study was approved by the Ethics Committee of Tongji Medical College, Huazhong University of Science and Technology on November 20, 2019. Written consent was obtained from the patient’s legal guardians.

SPSS statistical package program (SPSS 19.0 version; SPSS Inc., Chicago, IL, USA) was used for statistical analysis. The categorical data were analyzed using the chi-square (*χ*^2^) test, and the continuous data were analyzed using Student’s *t* test. Fisher’s exact test was used under those circumstances with fewer subjects in groups of interest. Data were presented as mean ± SD (range), median (range), or *n* (%). *P* < 0.05 was considered significantly different.

## Results

As shown in Table 1, 55 patients, including 33 males and 22 females, were included in the EF group, whereas 37 patients, including 21 males and 16 females, were included in the ESIN group (*P* = 0.45). The average age of patients in the EF group was 9.0 ± 2.8 years, and that of ESIN was 9.1 ± 2.9 years (*P* = 0.66). Patients in both groups were followed up for at least 2 years, with an average of 2.4 years (2–3 years). There was no significant difference between the two groups concerning sex, age, body weight, duration from injury to surgery, GA classification, and concomitant injuries.

**Table 1:** Patient demographics

Parameters		EF (*N* = 55)	ESIN (*N* = 37)	*P* value
Sex	Male	33 (60.0%)	21 (56.8%)	0.45
	Female	22 (40.0%)	16 (43.2%)	
Age (years)		9.0±2.8	9.1±2.9	0.66
Body weight (kg)		27.9±4.8	28.4±5.2	0.56
Side	Left	27 (49.1%)	19 (51.4%)	0.86
	Right	28 (50.9%)	18 (48.6%)	
From injury to surgery (h)		4.1±1.5	4.3±1.3	0.50
Gustilo-Anderson Classification	II	31 (56%)	21 (56.7%)	0.48
	IIIA	24 (44%)	16 (43.3)	
Concomitant injuries		35 (64%)	27 (72%)	0.19
Presence of fibular fracture		20 (36%)	14 (37.8%)	0.15

*EF* external Fixator, *ESIN* elastic stable intramedullary nail

Concomitant injuries: head, thoracic and abdominal, pelvic injuries

As shown in Table 2, there was no case of nonunion and malunion in either group. Three patients in the EF group suffered refracture after the hardware removal, but there was no case of refracture in the ESIN group.

**Table 2:** Clinical outcomes

Complication		EF (*N* = 55)	ESIN (*N* = 37)	*P* value
Malunion		0	0	> 0.99
Non-union		0	0	> 0.99
Deep infection		0	0	> 0.99
Refracture		3 (5%)	0	0.100
Implant prominence		0	6 (16%)	
Angulation (degree)	Coronal	3.4±1.4	1.9±1.2	0.02*
	Sagittal	5.7±3.1	4.6±3.1	0.04*
Wound infection		3 (5.5%)	2 (5.4%)	0.69
Pin tract infection		20 (36%)		
Hardware removal (weeks)		12.7±3.6	24.9±4.8	< 0.01*
Radiological union (weeks)		9.0±2.2	7.0±0.9	0.03*
LLD (mm) at last follow-up		12.1±4.4	7.3±4.3	< 0.01*

Major complications: loss of reduction, non-union, refracture

Minor complications: implant prominence, mild angulation, superficial infection

*LLD* limb length discrepancy

*< 0.05

The incidence of implant prominence was 16% in the ESIN group. The angulation was higher in the EF group than the ESIN group in both planes (*P* < 0.01). Besides, the angulation in the coronal plane was less than 5 degrees and in the sagittal plane was less than 10 degrees in both groups.

The incidence of wound infection showed no significant difference between the two groups. The radiological union was faster in the ESIN group (7.0 ± 0.9 weeks) than those in the EF group (9.0 ± 2.2 weeks) (*P* < 0.01). The LLD was more evident in the EF group (12.1 ± 4.4 mm) than in the ESIN group (7.3 ± 4.3 mm) (*P* < 0.01).

## Discussion

ESIN proved to be better in selected patients with open tibial shaft fractures and provided satisfactory clinical outcomes with relatively fewer complications than EF. However, ESIN required secondary surgery for hardware removal.

Tibial shaft fractures in children usually are not complicated and are treated with closed reduction and casting [13, 14]. However, in patients with open fractures, and compartment syndrome, surgical intervention is usually recommende d[14]. Still, some authors recommended manipulation followed by casting for open tibial fractures in children who did not require vascular reconstructio n[15]. In their study of Jones et al. [15], 16 fractures (19%) were treated using an external fixator and 65 (78%) using a cast. The average time to union was 15.5 weeks (range 9–31 weeks) for those treated with a frame and 10.4 weeks (range 5–40 weeks) for those treated with a cast. However, the malunion rate was not meticulously measured and analyzed. In another study reported by Charalambous et al in 2005 [7], 30 patients had manipulation and casting, and 9 patients had surgical internal or external fixation. There were 2 cases of infection in the cast-treated group and 2 in the surgical fixation group (*P* = 0.17). None of the fractures required a secondary surgical procedure to promote bone union. Three of the fractures were treated by manipulation and casting displaced, 2 required re-manipulation and casting, and 1 was converted to external fixation. This study was a retrospective study with a small cohort, and only 9 patients were included in the surgical group. Besides, surgical stabilization for open fracture is gaining popularity recently as it allows early mobilization and provides better alignment [5]. Therefore, for GA II and IIIA tibial shaft fracture, surgical stabilization was adopted in our hospital.

EF has been reported as a simple and effective choice for open tibial fracture [16]. There are many available construct designs, including circular fixator [17], monolateral fixator and hybrid external fixator [18], hexapod [19], and externalized locking plate [20]. Monolateral external fixator is the popular choice in our hospital. However, PTI, nonunion, loss of reduction, and refracture are known complications [21]. Therefore, another instrument modality was explored.

ESIN has been reported in the treatment for open tibial fractures in children, however, it also has various complications, including infection, delayed union, and angulation [10, 11, 22]. Pandya et al. published a comparative study of ESIN for patients with closed or open fractures in 2012 [10], and they enrolled 14 patients with open fractures and 12 patients with closed injuries as the control group. In their study, there was no statistically significant difference (*P* = 1.0) in terms of complications, including the rates of wound infections between the open (7.0%) and closed (4.0%) fractures groups. They did not report any cases of wound breakdown or osteomyelitis postoperatively. However, there was an increased rate of delayed union in the open fracture group (21.0% vs. 4.0%) (*P* = 0.02). Economedes DM et al. published an article in 2013 [22], and they included 17 patients with closed injuries and 21 patients with open fractures. In their study, open fractures treated with titanium elastic nails showed a significantly longer time to union requiring additional operative procedures and resulted in longer hospital stays. Of all the studies, there was no report of ESIN for the patient with Gustilo-Anderson Type III B and C injuries. Therefore, only external fixator is applied in our hospital for patients with severe open injuries, consistent with a previous study [23].

In patients with limited contamination such as GA grades I and II, a minimal invasive technique of locking plate has also been reported [24, 25]. However, it is unorthodox, and the removal of the plate is more troublesome than ESIN.

At our institute, for children with GA grade I open tibial fracture, casting followed debridement was preferred if the fracture is stable. And, ESIN was adopted if the fracture is unstable. EF was only reserved for GA grade II and III injuries.

In our study, there was no case of serious deep infection requiring secondary surgery in either group, possibly due to aggressive debridement and timely administration of antibiotics.

The incidence of implant prominence was a troublesome complication, possibly caused by painful bursitis around the entry point [26]. However, most of our patients were able to tolerate it. Superficial infections were mostly around the pin tract in the EF group, and all of them could be managed easily with oral antibiotics.

None of our patients had deep infections or osteomyelitis in either group which was possibly due to aggressive debridement and appropriate administration of antibiotics. Similarly, postoperative coronal and sagittal plane angulation was more common with the EF group, however, it was within an acceptable range, and the result was consistent with previous reports [27]. Moreover, the radiological union was faster in the ESIN group in our study, which might be caused by the micro-motion in the fracture site leading to the stimulation of bone formation [28].

LLD is a common complication in pediatric long bone fracture especially in the femur and tibia [29, 30]. In our study, it was more evident in the EF group than the ESIN group, consistent with a previous report [27]. However, none of the patients had significant LLD in either group.

There were 3 cases of refracture around the Schanz pin site in the EF group. Fracture usually occurred 4–6 weeks after the removal of hardware, and they were all managed conservatively. Moreover, there was no case of refracture in the ESIN group. Patients in the ESIN group were disadvantaged by the requirement of secondary surgery for implant removal, while the EF was removed during out-patient visits.

Our study has the following inherent limitations. The allocation process of patients to either ESIN group or EF group depended on the preference of the surgeon in charge and this strategy may cause allocation bias. Therefore, our findings should be interpreted with caution. Furthermore, cost-effectiveness remains to be investigated.

## Conclusion

ESIN is a viable option in selected patients of GA grade II and IIIA open tibial fractures with comparable clinical outcomes as external fixator. Pin tract infection is the most troublesome complication in the EF group while implant prominence is a nuisance in the ESIN group.

## Data Availability

The data sets supporting the conclusion of this article are included within the article. Upon request, raw data can be provided by the corresponding author.

## Abbreviations

ESIN: Elastic stable intramedullary nail
EF: External fixation
LLD: Limb length discrepancy
GA: Gustilo-Anderson
